# Transoral Endoscopic-Assisted Resection of Laryngeal Schwannoma: A Case Report 

**DOI:** 10.22038/IJORL.2023.71324.3427

**Published:** 2024-01

**Authors:** Dian Paramita Wulandari, Anisa Haqul Khoiria, Elida Fadhilatul Latifa

**Affiliations:** 1 *Department of Otorhinolaryngology Head and Neck Surgery, Faculty of Medicine, Public Health and Nursing, Universitas Gadjah Mada, Yogyakarta, Indonesia.*

**Keywords:** Laryngeal schwannoma, Transoral approach, Surgical management, Tracheostomy

## Abstract

**Introduction:**

Schwannoma, a peculiar benign nerve sheath tumour, is frequently hard to differentiate from other nerve tumours, such as neurofibroma. Around 25%-45% of all schwannomas emerge in the head and neck region, but only 0.1-1.5 % involve laryngeal structure. This tumour is most accurately diagnosed with biopsy via direct laryngoscopy; however, at some points this approach cannot detect a definitive diagnosis due to the surrounding capsule of the tumour and its similar histopathologic finding with other nerve sheath tumour.

**Case Report:**

Here, a case of 56-year-old female is reported with chief complaints of severe progressive dyspnea and dysphagia. Diagnosis of schwannoma was confirmed on radiological and histopathological examination with certain hurdles. A complete surgical excision via endoscopic approach was done, revealing that the bottom of the mass was attached to the right arytenoid mucosa. The histopathological features showed non-malignant atypical neurofibroma but later confirmed as laryngeal schwannoma from immunohistochemical staining.

**Conclusion:**

Although schwannoma has an excellent outcome and prognosis when occurring elsewhere in the body, laryngeal involvement is an extremely rare area for this lesion. Complete resection with a patient-customized approach to the lesion is required to avoid relapses and provide good functional results.

## Introduction

Larynx neurogenic tumours are rare, contributing to a mere 0.1-1.5% of all benign laryngeal lesions.^1 ^Two types of neurogenic tumours that must be differentiated are schwannoma and neurofibroma. Schwannoma originates from Schwann cells located perineural, distinctively encapsulated, and growing near the parental nerve. Neurofibroma, however, is a derivate of perineural fibrocytes, not encapsulated, and commonly conjoined with the fascicles of parental nerve. Location of both these types within the larynx is rare, with schwannoma becoming slightly more common than neurofibroma; 80% are found in the aryepiglottic fold and 20% in the true or false vocal cords. The superior laryngeal nerve internal branch is most likely the origin of this nerve lesion (1,2).

## Case Report

**Fig 1:**
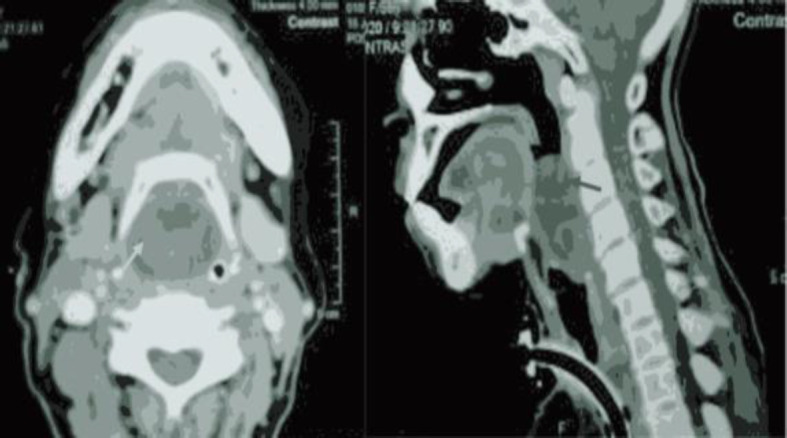
A computed tomography (CT) scan of the neck shows a round mass obstructing the larynx from the axial view (yellow arrow) and sagittal view (red arrow).

A 56-year-old female attended the ENT outpatient clinic at DR Sardjito Public Hospital with progressive throat discomfort and dysphagia for over five years with a previous history of severe dyspnoea due to upper airway obstruction. An emergency tracheostomy was done in the regional hospital. The patient was then referred to our hospital for further management. Upon initial assessment, the patient could not swallow adequately; therefore, we inserted a nasogastric tube to fulfil the nutritional intake. Laryngeal endoscopy revealed a smooth-surfaced, well-defined mass obstructing the laryngeal inlet and oesophageal introitus. Computed tomography (CT) imaging with contrast showed a well-circumscribed 4.8x3.4x2.1 cm cystic mass at the hypopharyngeal region at the level of the second and fourth cervical vertebrae that completely obstruct the upper airway passage (Figure 1). 

**Fig 2:**
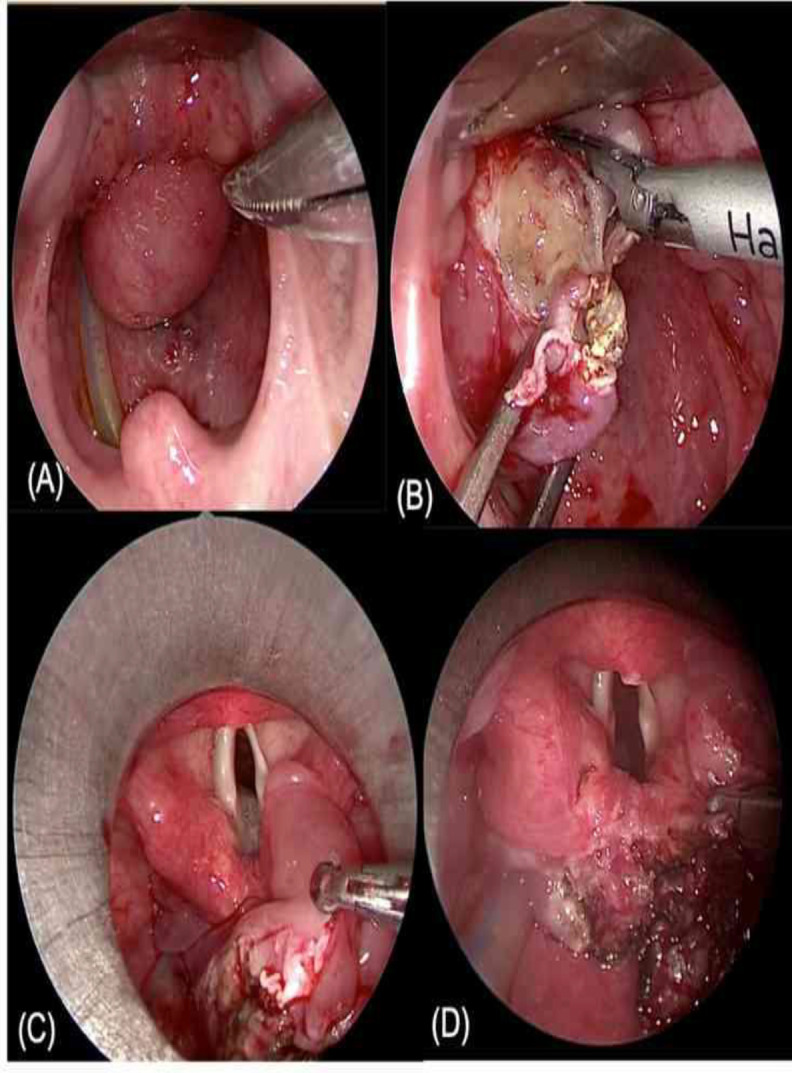
Direct laryngoscopy showed: (A) Smooth-surfaced tumour mass seen as a bulge that obstructs the sight of vocal cords; (B) Debulking incision to assess the base of the mass; (C) Exposure of the mass attachment to the right aryepiglottic mucosa; (D) Complete excision of the mass using the harmonic scalpel

An endoscopic tumour excision via transoral direct laryngoscopy was settled to determine the base of the mass and for diagnostic histopathology. The procedure was done under general anaesthesia via a tracheostomy tube. Intraoperatively, the base of the mass was attached to the right arytenoid mucosa. However, we ensured that the other part of the mass surface was not attached to the following posterior and lateral laryngeal walls, epiglottis, or oesophageal introitus. It is challenging to remove the whole integrated mass. Therefore, we performed initial debulking followed by excision of the remaining tumour mass using an ultrasonic harmonic scalpel (Figure 2).

**Fig 3:**
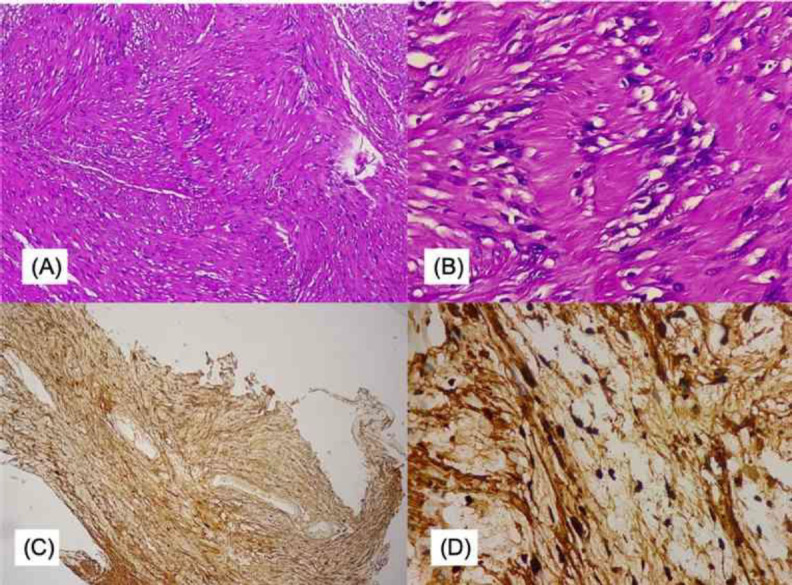
Histologic results of the laryngeal schwannoma. (A) The spindle cells were placed in compact bundles with the nuclei located as a palisade from low magnification. (B) The cells are partly dispersed with foci arranged loosely. The schwannoma cells are strongly immunoreactive for S-100 protein. (C&D) 4x and 40x histological picture of the Schwannoma: The tumour cells show strong and diffuse staining for S100 protein

No complications were found, and the patient was sent home three days following the procedure. Histopathologic investigation of the tumour showed the features of non-malignant atypical neurofibroma; the result was later confirmed using S100 immunohistochemistry staining that confirmed the diagnosis of laryngeal schwannoma (Figure 3).

**Fig 4:**
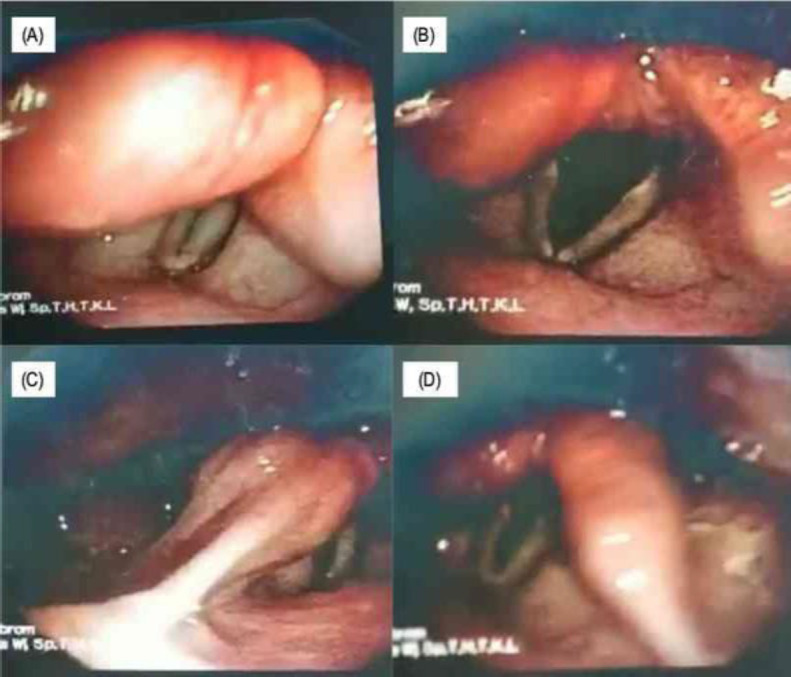
Flexible laryngoscope evaluation on 12^th^ weeks after surgery: (A)&(B) Improvement of the right vocal cord mobility, no remaining mass nor sign of recurrence in the laryngeal inlet with patent upper airway; (C)&(D) Absence of tumour recurrence in the right and left arytenoid mucosa

Laryngeal endoscopic evaluation was done periodically at 1^st^, 2^nd^, 4^th^, 8^th^, and 12^th^ week after surgery. Voice and swallowing therapy was given as early as possible, starting two weeks after surgery. The patient underwent decannulation and nasogastric tube removal in the fourth week after surgery with good swallowing function, no dyspnoea, no abnormalities in vocal cord motions, and no vocal ability after the 12^th^-week post-surgery (Figure 4). The patient was subsequently examined for two years without any relapse.

## Discussion

A benign neurogenic tumor of the larynx, constituting a small percentage (0.1 to 1.5%) of all benign laryngeal tumors, can be classified into schwannoma and neurofibroma. Schwannomas originate from Schwann cells from perineural cells, growing externally to their parent nerve fascicles and can invade various somatic or sympathetic nerves in the body, excluding the olfactory and optic nerves lacking Schwann's cells (3,4).

Neurofibromas arise from perineural fibrocytes, including nerve fibers and sheath cells, typically conjoined with the nerve trunk (4,5). This distinction is crucial in surgery, as the excision of a tumor originating from a nerve is theoretically feasible in schwannomas, unlike in neurofibromas (5). Malignant transformation is rare in schwannomas but reported in 10% of neurofibromas. Laryngeal schwannomas most frequently occur in the supraglottic area, specifically in the aryepiglottic fold or the true or false vocal cords (5,6).

Schwannoma occurs at all ages, with a higher incidence in the fourth and fifth decade of life, more commonly in females. Several studies indicate a higher likelihood of laryngeal schwannomas occurring in females. This condition is typically observed in individuals aged between 20 and 50 years, although there have been instances reported in individuals as young as eight. Due to its slow growth, patients may not experience symptoms until the tumor reaches a size that begins to affect nearby structures, leading to breathing, swallowing, speech, and general discomfort in the throat. The most frequent findings are hoarseness, as well as dysphagia, dyspnoea, and foreign body sensation (7).

Computed Tomography (CT) and Magnetic Resonance Imaging (MRI) are intended to determine the primary site and extension. The choice between CT and MRI usage relies on the accessibility of each imaging method and the level of detail needed for soft tissue characterization. CT effectively outlines the tumor's extension but has limitations in soft tissue characterization. In contrast, MRI offers superior soft tissue characterization (6).

Normally, the anomaly exhibited clear boundaries, adopting a round or oval shape, showing attenuation with muscle, and frequently displaying heterogeneous enhancement. However, it is challenging to make distinctions between schwannoma and neurofibroma through radiological means because they share similar distinctive characteristics (8,9).

Because endoscopic evaluations and imaging studies cannot conclusively eliminate the possibility of malignancy, the definitive diagnosis of schwannoma must rely on histological examination. Three well-established histological criteria guide schwannoma diagnosis: the existence of a capsule, the finding of stromal Antoni A (compressed, bipolar cells with nuclei arranged in a palisade form), and/or Antoni B (spindle cells loosely arranged inside a myxoid matrix) patterns, and the confirmation of the positive result of S-100 protein (9). All of these features were present in our case study. Otherwise, neurofibromas are not encapsulated and are made of various cell types with elongated spindle cells intertwined with axons and collagen fibres (6,9).

The primary treatment for laryngeal schwannoma is surgical intervention, with the possibility of requiring a tracheostomy to manage the airway. The choice of surgical method relies on the size and location of the lesion. Smaller ones can be addressed through endoscopic methods, with or without a laser, and transoral CO2 laser microsurgery is a viable option. For larger tumors, a tracheostomy may be completed by an external approach, such as lateral thyrotomy, lateral pharyngotomy/ cervicotomy, or the laryngofissure technique. 

It is crucial to perform a wide excision to avoid recurrence, as swift relapse can occur in months if the schwannoma is incompletely resected. Reports indicate restoration of vocal cord mobility following surgery, irrespective of the chosen approach (10). 

Our case presented a massive bulky tumour that obstructed the upper airway, resulting in the need for an emergency tracheostomy prior to the surgical excision. According to previous case studies in most literature, such a large mass requires an external approach. However, we performed a transoral endoscopic approach to our patient rather than anterior or lateral cervicotomy in order to avoid external tissue damage and the appearance of an external surgical scar. Another circumstance for us to consider this approach was due to the tumour location that we anticipated was very imminent to the oesophageal introitus. Using this approach, we could carry out a complete excision of the mass with a harmonic scalpel without damaging the surrounding tissue. There was an improvement in the right vocal cord movement in the 12^th^ week after surgery with no sign of recurrence. Tracheostomy is possibly required preoperatively in the occurrence of airway blockade or after the procedure to prevent life-threatening complications. In cases necessitating an open procedure, tracheostomy was carried out in 60% of instances, whereas patients managed with endoscopy for schwannomas of recurrent laryngeal nerve never underwent tracheostomy (11).

In our case, the tracheostomy has been done as an emergency procedure for lifesaving purposes due to complete airway obstruction.

In a prior case study conducted by Mariani et al. (2020), they employed *en bloc* excision using a transoral-trans pharyngeal CO2 laser to address a sizable schwannoma located in the intralaryngeal segment of recurrent laryngeal nerve. This case differs from ours in the need for an initial emergency tracheostomy and tumour size and location. 

The tumour appeared bigger in our case and much more obstructive to the airway. It also appeared as a submucosal bulging visible from the hypopharynx area, whereas in the case of Mariani et al. (2020), the tumour was embedded in the pyriform sinus and caused only partial upper airway obstruction. Therefore, endotracheal intubation was still sustainable. The lack of improvement in the left vocal cord paralysis, positioned centrally, throughout the follow-up indicates that the tumor likely originates from the intralaryngeal section of the recurrent nerve (11). 

Another previous case report by Wang et al. (2014) performed a trans-oral resection using a micro laryngoscope for the tumour located in the aryepiglottic fold with quite a large dimension of 5.8x2.9x2.6 cm; post-operative tracheostomy could be avoided. The result from a short-term follow-up of the case was satisfactory. 

Age and symptoms considerations are the reason behind the choice of surgical approach; also, due to the slow-paced growth of the schwannoma itself, the excision under micro laryngoscope without tracheostomy was considered plausible (12). Conversely, Sudhakar et al. (2011) conducted a case study involving elective tracheostomy followed by lateral pharyngotomy to remove a large laryngeal schwannoma attached to the left aryepiglottic fold extending into the left pyriform sinus. Immediate postoperative decannulation was challenging due to significant paraglottic space, leading to laryngeal edema and upper airway obstruction. 

This approach was chosen based on the tumor's size exceeding 5 cm and occupying the entire supraglottic region, believing an external approach could extract the mass without disrupting the laryngeal framework (13) Tse & Anwar (2015) similarly employed an external approach via laryngofissure in their case study, performing excision of a supraglottic schwannoma. 

Tracheostomy ensured airway security until histological results were available. The procedure resulted in uneventful removal of the 5.5x3.0x2.0 cm mass, with no recurrence noted after a 2-month follow-up. This approach was chosen due to the challenging accessibility of the tumor mass through micro-laryngoscopy (14).

## Conclusion

Extracting obstructive laryngeal schwannoma from the arytenoid area poses a challenge for head and neck surgeons, requiring customized planning based on individual case requirements. 

This study assessed the clinical results of patients with laryngeal schwannoma underwent endoscopy-assisted resection via the transoral approach, utilizing an ultrasonic harmonic scalpel. The procedure demonstrated a recurrence-free larynx and favorable functional outcomes.
